# Effect of a Mobile Health App on Adherence to Physical Health Treatment: Retrospective Analysis

**DOI:** 10.2196/31213

**Published:** 2021-12-02

**Authors:** Jay Greenstein, Robert Topp, Jena Etnoyer-Slaski, Michael Staelgraeve, John McNulty

**Affiliations:** 1 Kaizenovate Kaizo Clinical Research Institute Kaizo Health Rockville, MD United States; 2 University of Toledo Toledo, OH United States

**Keywords:** adherence, self-discharge, phone app, physical therapy, chiropractor, mobile phone

## Abstract

**Background:**

Adherence to prescribed medical interventions can predict the efficacy of the treatment. In physical health clinics, not adhering to prescribed therapy can take the form of not attending a scheduled clinic visit (no-show appointment) or prematurely terminating treatment against the advice of the provider (self-discharge). A variety of interventions, including mobile phone apps, have been introduced for patients to increase their adherence to attending scheduled clinic visits. Limited research has examined the impact of a mobile phone app among patients attending chiropractic and rehabilitation clinic visits.

**Objective:**

This study aims to compare adherence to prescribed physical health treatment among patients attending a chiropractic and rehabilitation clinic who did and did not choose to adopt a phone-based app to complement their treatment.

**Methods:**

The medical records of new patients who presented for care during 2019 and 2020 at 5 community-based chiropractic and rehabilitation clinics were reviewed for the number of kept and no-show appointments and to determine whether the patient was provider-discharged or self-discharged. During this 24-month study, 36.28% (1497/4126) of patients seen in the targeted clinics had downloaded the Kanvas app on their mobile phone, whereas the remaining patients chose not to download the app (usual care group). The gamification component of the Kanvas app provided the patient with a point every time they attended their visits, which could be redeemed as an incentive.

**Results:**

During both 2019 and 2020, the Kanvas app group was provider-discharged at a greater rate than the usual care group. The Kanvas app group kept a similar number of appointments compared with the usual care group in 2019 but kept significantly more appointments than the usual care group in 2020. During 2019, both groups exhibited a similar number of no-show appointments; however, in 2020, the Kanvas app group demonstrated more no-show appointments than the usual care group. When collapsed across years and self-discharged, the Kanvas app group had a greater number of kept appointments compared with the usual care group. When provider-discharged, both groups exhibited a similar number of kept appointments. The Kanvas app group and the usual care group were similar in the number of no-show appointments when provider-discharged, and when self-discharged, the Kanvas app group had more no-show appointments compared with the usual care group.

**Conclusions:**

Patients who did or did not have access to the Kanvas app and were provider-discharged exhibited a similar number of kept appointments and no-show appointments. When patients were self-discharged and received the Kanvas app, they exhibited 3.2 more kept appointments and 0.94 more no-show appointments than the self-discharged usual care group.

## Introduction

### Background

In health care, adherence has been defined as “the extent to which a person’s behavior corresponds with the recommendations from a healthcare provider” [1] and is the primary determinant of treatment success [1]. When the prescribed medical treatment involves physiotherapy to treat chronic musculoskeletal pain, adherence to the prescribed therapy has been reported to be critical for the successful resolution of the problem [2]. Low adherence to prescribed treatment has been identified as a challenge among many health care disciplines, including physiotherapy. Maintaining adherence to prescribed medical treatment is essential to facilitate maximum recovery following an injury and promote optimal health [3]. Sluijs et al [4] reported that between one-third and two-thirds of patients involved in treatment programs that included physiotherapy are not adherent with the prescribed treatment plan. A component of not adhering to prescribed medical treatment in primary care is not attending scheduled clinic appointments. When a patient prematurely terminates treatment against the advice of the provider, it is termed *self-discharge* as compared with the patient completing their prescribed treatment, which is termed *provider-discharged*. Not attending a single scheduled clinic appointment is termed a *no-show appointment* and is defined as an appointment in which the patient did not present for treatment or did not contact the clinic to cancel the appointment [5]. Both self-discharge and no-show appointments reduce revenue, result in suboptimal use of clinical and administrative staff, may lengthen wait times for patients, and negatively affect the continuity of care [6]. In primary care, the rate of no-show appointments ranges from 19% [7] to 42% [8] and is estimated to cost the US health care system US $150 billion per year [9]. Moore et al [10] reported that no-show appointments negatively affected 25% of scheduled time in a family medicine clinic and resulted in a loss of 14% of the anticipated daily revenue. Patients with frequent no-show appointments experienced worse health care outcomes [3]. In a nationwide survey of physical therapists, investigators reported that 10.4% of their patients’ appointments were no-show appointments in private clinics, which was significantly lower than the percentage of patients who were no-show appointments in hospital campus clinics (14.53%) [11]. This low adherence to physiotherapy treatment has not changed over the past 27 years [11]. Other investigators have reported adherence rates with prescribed physiotherapy to be as low as 37.6% [12]. Thus, a primary explanation for the less-than-expected impact of physiotherapy in treating chronic musculoskeletal problems [13] may be a lack of adherence to the prescribed therapy by the patients and not the efficacy of the prescribed physiotherapy.

A variety of procedures have been introduced in outpatient clinics in an attempt to reduce the problem of self-discharge and no-show appointments. Providers have introduced different methods to reduce no-show appointments, including reminder procedures or penalizing the patient financially for a no-show appointment. The efficacy of these methods has not been clearly determined. Satiani et al [14] reported that automated reminder systems did not significantly reduce the rate of no-show appointments. Other investigators found no effect [15] or only moderate effects [7] of automatic reminder systems to reduce no-show appointments. However, when appointment reminders were from actual clinic staff, the no-show rate was significantly reduced [16]. A continuous quality improvement study by Teo et al [17] indicated that reminders from an actual person resulted in lower no-show appointments (3%) when compared with message or voice mail reminders (24%). In a randomized controlled trial (RCT) where physical therapy patients received clinic appointment reminders sent to their cell phone, the no-show appointment rate was lower (11%) compared with patients who did not receive an appointment reminder (16%) [18]. A comprehensive review of the literature concluded that reminder interventions, including telephone, mail, SMS text messaging, and email reminders, all moderately reduced no-show outpatient clinic appointments [19]. This finding is consistent with a more recent meta-analysis of the literature that concluded that patients who received a text-based electronic notification of an upcoming health care appointment were 25% less likely to no-show for their appointment [20]. Penalizing or imposing a financial charge on patients for no-show appointments has been proposed as an effective approach to reducing this problem by economists [21]. However, a large empirical study did not demonstrate the efficacy of imposing a financial charge on no-show appointments to reduce future no-show appointments among outpatients [22]. Reminder procedures or penalizing the patient financially for no-show appointments have not consistently demonstrated reductions in no-show appointments.

A number of recent studies have presented evidence that supports the feasibility, acceptability, and efficacy of digital health interventions in treating different chronic medical conditions. In addition to providing text-based messaging about upcoming health care appointments, mobile phone apps have been designed to promote patient engagement in their care, including improving self-care and adherence to prescribed health care therapies. In a review of 279 commercially available mobile phone apps to manage pain that included education, self-monitoring, social support, and goal setting, the authors concluded that the efficacy of most apps was not supported by empirical research [23]. A more recent review of 15 studies evaluating the effects of phone-based apps involving pain management concluded that these apps are workable, well-liked by patients and health care professionals, and can result in reductions in pain [24]. In a more recent study, Huber et al [25] reported that a multidisciplinary phone-based app to manage pain, Kaia, including prescribed exercises, education, relaxation exercises, and coaching, resulted in statistically and clinically significant reductions in pain. MacIsaac et al [26] examined an innovative, smartphone app–based resilience intervention—the JoyPop app—introduced among first-year undergraduate students. After using the app at least twice daily for 4 weeks, 156 participants reported improved emotional regulation and depression. This positive impact of the JoyPop app was directly related to the frequency of using the app. Irvine et al [27] studied a mobile web intervention called *FitBack* that was designed to encourage users to adopt cognitive and behavioral strategies based on social cognitive theory and the theory of planned behavior to support their self-efficacy to engage in prescribed pain management and prevention behaviors. The findings of this study demonstrated that the standalone mobile web intervention that tailored content to users’ preferences and interests was an effective tool for self-management of lower back pain. The researchers concluded that there is considerable value in this type of intervention as a potentially cost-effective tool that can reach large numbers of patients to encourage adherence to prescribed medical treatment [27]. More recently, electronic medical record (EMR)–tethered patient portals have become available on phone-based apps. In a study of 957 patients who accessed an EMR-tethered portal, participants reported positive experiences and decreases in health system use and exhibited fewer no-show appointments [28]. The authors of a retrospective, observational study of 46,544 primary care patients reported that adoption, use, and benefits of using EMR-tethered portals available on a phone app were not clearly linked. However, these authors concluded that patients who used the messaging and laboratory functions of the app were less likely to exhibit no-show appointments compared with other user subgroups [29].

In addition to these individual trials, a number of review articles support the positive impact of technology-based health interventions. Ramsey et al [30], after their review of 21 peer-reviewed journal articles, reported the efficacy and increasing access to digital technologies, including eHealth and mobile health (mHealth), may improve the mental and physical health of youth undergoing cancer treatment and survivors of childhood cancer. Following a systematic review, Badawy et al [31] concluded that mobile phone app interventions could improve medication adherence among adolescents with chronic health conditions, and the current literature indicates that these mobile phone app interventions are feasible and accepted by adolescents, and there is modest evidence to support the efficacy of these interventions. These findings are consistent with those of Oikonomidi et al [32], who conducted a systematic review of mHealth behavior change interventions (SMS text messages and smartphone apps) in RCTs. After reviewing 231 RCTs, the authors concluded that mHealth behavior change interventions lack information that would be useful for providers, including the long-term impact of the interventions’ health outcomes and information needed for replication of the RTC. Finally, Shah and Badawy [33] provided a systematic evaluation of the feasibility, accessibility, satisfaction, and health outcomes of telemedicine services among pediatric populations with different health conditions. After reviewing 11 articles in this area, the authors concluded that telemedicine services for the general public and pediatric care are comparable with or better than in-person services. Although promising, technology-based health interventions, including mobile phone apps designed to support adherence to prescribed medical treatment, have not been extensively studied on adherence to outpatient physical health treatment.

### Purpose

This study aims to compare adherence to prescribed physical health treatment among patients attending a chiropractic and rehabilitation clinic who did and did not choose to adopt a phone-based app to complement their treatment.

### Hypotheses

Hypothesis 1: Patients receiving physical health treatment who choose to receive the phone-based app compared with physical health patients who choose not to receive the phone app will exhibit greater rates of completing their prescribed therapy (fewer self-discharge and greater provider-discharge).

Hypothesis 2*:* Patients receiving physical health treatment who choose to receive the phone-based app compared with physical health patients who choose not to receive the phone app will exhibit fewer no-show appointments and more kept appointments.

### Research Question

Research question 1: Does self-selecting to receive the phone-based app or not and being self-discharged versus provider-discharged differentially affect no-show and kept appointments among patients prescribed physical health treatment?

## Methods

### Design

A retrospective analysis of all new outpatient medical records from a multisite physical health practice was performed between January 2019 and December 2020. Beginning in January 2019, all new patients admitted to this practice were offered the opportunity to download a phone-based app, the Kanvas app, during their initial visit to complement their treatment. New patients who downloaded and registered on the phone-based app self-selected into the Kanvas app group. New patients admitted to this physical health practice during this same time who did not download and register on the app self-selected into the usual care group. Each patient’s medical record was accessed 4 months after their initial visit to determine whether they prematurely terminated treatment against the advice of the provider (self-discharged) or if they completed their prescribed treatment (provider-discharged). The number of no-show appointments and the number of kept appointments were also extracted from each patient’s medical records. This resulted in a quasi-experimental, 2-group design in which the records of all patients initially presenting for treatment between January 2019 and December 2020 were reviewed and included in the analysis.

### Sample

The medical records of new patients who presented during the study period for care at 1 of 5 community-based physical health clinics in the Greater Washington DC area (n=4203) were initially screened as participants in this study. These clinics specialize in treating pain and increasing functional abilities. During the initial visit, all patients were informed that they could download a mobile app on their phone that they could use to complement the care they were receiving at the clinic. At this time, all patients were told about the components of the app and the reward structure as a result of using the app. Patients were also told that the use of the app was voluntary and would in no way affect their care or relationship with their provider or the clinical agency. Patients were excluded from the study if, following their initial visit, they were referred to another medical clinic for care, were employed by one of the targeted clinics, or died before completing therapy (77/4203, 1.83%). This record review study was approved by the Sport and Spine Rehab Clinical Research Foundation institutional review board number SSR.2021.1.

### Procedure

**Figure 1:**
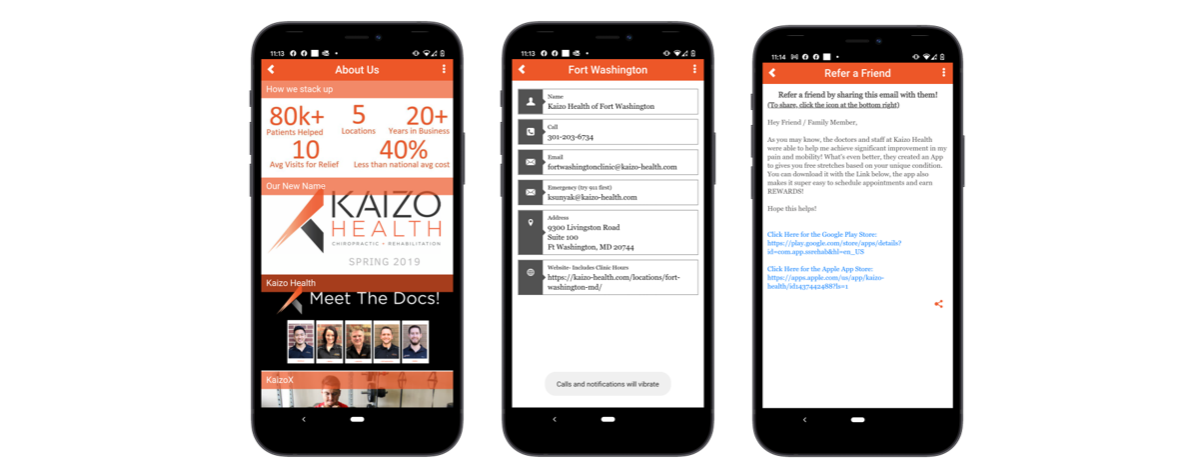
Tiles from the Kanvas app.

**Figure 2:**
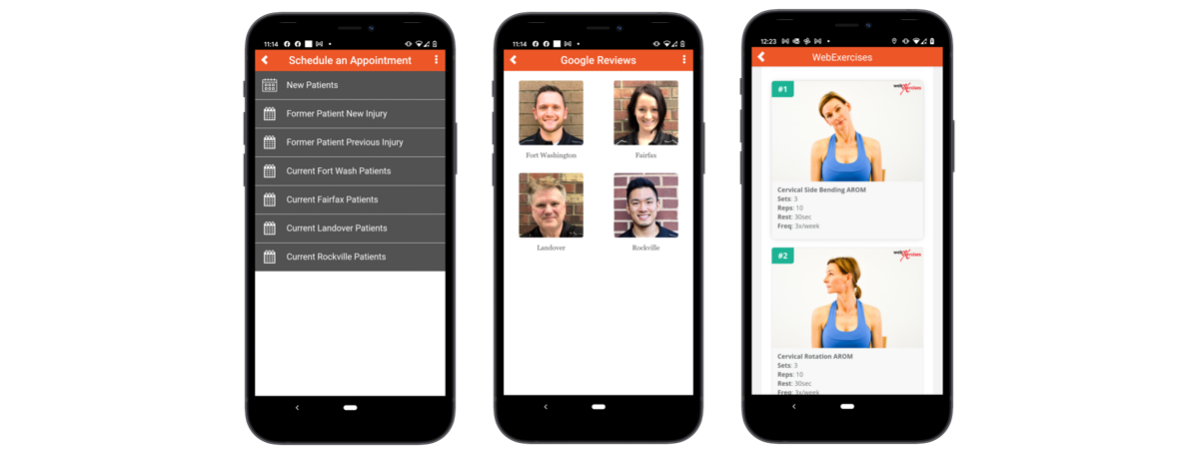
Additional tiles from the Kanvas app.

**Figure 3:**
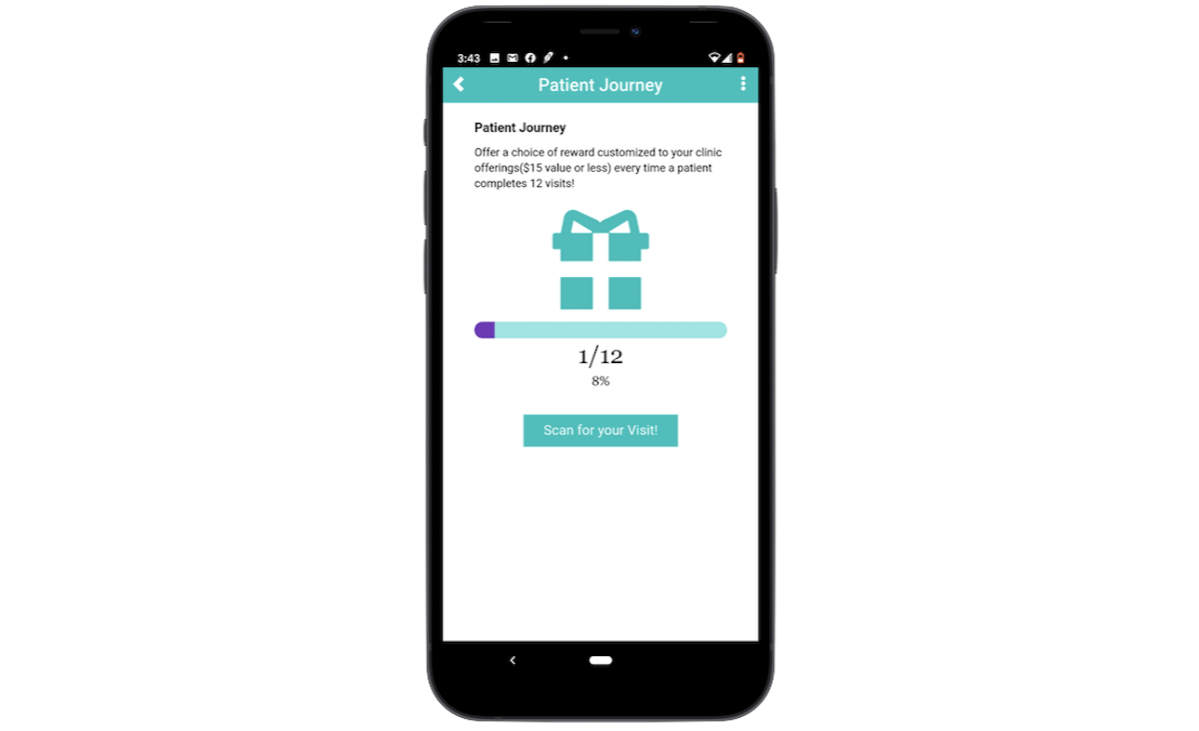
Kanvas app patient journey.

During the initial visit at one of the targeted clinics, each patient completed an initial assessment with a practitioner (physical therapist or chiropractor) who prescribed a plan of care that included home exercises and a series of follow-up clinic visits. This plan of care and the number and frequency of follow-up clinic visits were individualized to the type and severity of the patient’s condition. Patients were scheduled for their next follow-up visit during the initial visit and were informed that their account would be charged US $25 if they did not attend this scheduled visit or did not contact the clinic to cancel the appointment within 24 hours of the appointment (no-show appointment). The Kanvas app is a customized private practice app designed for patient engagement with their specific health care provider. The initial screen includes various *tiles* in which the patient can engage with the office. These tiles include *contact us*, *about us*, *refer a friend*, *request an appointment*, *review us*, and *home exercise* (Figures 1 and 2). In addition, a built-in gamification system, the *rewards* tile (Figure 3), was designed to reward the patient for attending their scheduled clinic appointments. This feature is Office of Inspector General compliant, offering an item as a reward valued at <US $15 once the patient completed 12 prescribed visits or was provider-discharged. This feature documented a running total of the number of clinic visits that the patient had attended. The feature is patient-directed, where they scan a QR code at the front desk of the clinic at every visit. When the patient reaches 12 prescribed visits or is provider-discharged, they are eligible for a reward.

### Outcome Variables

The medical records of all eligible patients who were initially seen in the targeted clinics over the 24-month duration of the study and were discharged from care were reviewed. On the basis of the discharge summary documentation on the patient’s EMR, patients were classified as completing prescribed therapy and being discharged by their provider (provider-discharged) or not completing their prescribed therapy and self-discharging themselves (self-discharged). In addition, the number of scheduled appointments they attended (appointments kept) and the number of scheduled appointments they failed to attend (no-show appointments) were extracted from each patient’s EMR.

### Analysis Plan

Data were extracted from the EMRs of all patients identified as eligible for the study and transcribed into a Microsoft Excel (Microsoft Inc) spreadsheet. These data were validated to include only eligible patients, and then individuals were grouped according to the Kanvas app group or usual care group and provider-discharged or self-discharged groups. As the study took place during 2020, when the COVID-19 pandemic was occurring, the analysis to address the hypotheses was conducted separately for both study years. The first hypothesis was addressed by calculating chi-square statistics to compare the proportion of the Kanvas app group or the usual care group participants who were classified as provider-discharged or self-discharged. The remaining outcome variables were all continuous, and to address the second hypothesis, separate repeated-measures analysis of variance (ANOVA) statistics were calculated with year (2019 vs 2020), group (Kanvas app vs usual care), and the interaction of year and group as independent factors to determine differences in no-show appointments or kept appointments. Significant main or interaction effects detected in any of these repeated-measures ANOVA statistics were further explored by calculating Bonferroni post hoc comparisons to determine differences between the means being compared. Finally, the research question was addressed by collapsing the data across both study years and then conducting a 2×2 factorial ANOVA of the outcome variables of no-show appointments and kept appointments. The independent factors in these factorial ANOVAs were the usual care group versus the Kanvas app group and self-discharged versus provider-discharged and the interaction of study group and discharge type. Significant main or interaction effects were further explored by calculating Bonferroni post hoc comparisons to determine the differences between the means being compared. The level or statistical significance for all analyses was set a priori at *P*<.05. A total of 4126 patients were included in this study, with 2629 (63.72%) choosing to receive the usual care and 1497 (36.28%) choosing to use the Kanvas app. This sample size, using the 2×2 factorial ANOVA statistic with type 1 error set at 0.05 and maintaining statistical power at 0.8 (1-β), would be able to detect a small effect size Cohen *d*=0.05 in no-show appointments or kept appointments between the 2 study groups.

## Results

### Description of the Sample

A total of 4203 patient records were reviewed, and 98.17% (4126/4203) were included in the analysis, with 49.1% (2026/4126) and 50.9% (2100/4126) of patients being initially seen in the targeted clinics in 2019 and 2020, respectively. In 2019, 69.2% (1402/2026) of the patients initially seen that year self-selected into the usual care group (mean age 40.38, SD 13.82 years), whereas this percentage significantly declined (*χ*^2^_1_=51.8; *P*<.001) to 58.42% (1227/2100) of the sample who were initially seen in the targeted clinics during 2020. Table 1 indicates that during 2019, 50.8% (317/624) of the Kanvas app group (mean age 38.31, SD 11.63 years) were provider-discharged, which was significantly greater than the 46.01% (645/1402) of the usual care group who were provider-discharged (*χ*^2^_1_=4.0; *P*<.046. This pattern was repeated in 2020 during the COVID-19 pandemic, with 38.4% (335/873) of the Kanvas app group being provider-discharged, which was significantly greater than the 31.38% (385/1227) of the usual care group being provider-discharged (*χ^2^*_1_=11.1; *P*<.001).

**Table 1:** Type of discharge by the Kanvas app group versus the usual care group.

Variables		2019				2020				Total
		Sample size	Self-discharge	Provider-discharge	Sample size		Self-discharge	Provider-discharge		
**Group, n (%)**										
	Usual care	1402 (100)	757 (53.99)	645 (46.01)	1227 (100)		842 (68.62)	385 (31.38)	2629 (63.72)	
	Kanvas app	624 (100)	307 (49.2)	317 (50.8)	873 (100)		538 (61.6)	335 (38.4)	1497 (36.28)	
	Total	2026 (100)	1064 (52.52)	962 (47.48)	2100 (100)		1380 (65.71)	720 (34.29)	4126 (100)	
**Test statistic within study year**										
	Chi-square (*df*)	N/A^a^	4.0 (1)	4.0 (1)	N/A		11.1 (1)	11.1 (1)	N/A	
	*P* value	N/A	.046	.046	N/A		<.001	<.001	N/A	

^a^N/A: not applicable.

### Results to Address Hypotheses and Research Question

Table 2 presents the means and SEs for the number of kept appointments and no-show appointments in the Kanvas app and the usual care groups in 2019 and 2020. This table indicates that the Kanvas app group kept a similar number of appointments compared with the usual care group in 2019 (10.20 vs 8.68); however, the Kanvas app group kept significantly more appointments than the usual care group in 2020 (11.63 vs 7.67). During 2020, the Kanvas app group exhibited 2.89 (SE 0.10) no-show appointments that were significantly greater than the number of no-show appointments exhibited by this group during 2019 (mean 1.89, SE 0.08) and significantly more than the no-show appointments by the usual care group during 2020 (mean 2.14, SE 0.08).

**Table 2:** Comparing kept and no-show appointments of the Kanvas app versus usual care groups by year.

Outcome measure	2019, mean (SE)		2020, mean (SE)			Statistical comparison: interaction effect	
	Usual care	Kanvas app	Usual care	Kanvas app	*F* test (*df*)		*P* value
Kept appointments	8.68 (0.22)	10.20 (0.33)	7.67 (0.24)	11.63 (0.28)^a^	20.28 (1, 4122)^b^		<.001
No-show appointments	1.96 (0.11)	1.89 (0.08)	2.14 (0.08)	2.89 (0.10)^a,c^	13.50 (1, 4122)^d^		<.001

^a^Indicates a significant difference between groups within a specific year.

^b^Bonferroni minimum significant difference=2.37.

^c^Indicates a significant difference within a group between study years.

^d^Bonferroni minimum significant difference=0.65.

**Figure 4:**
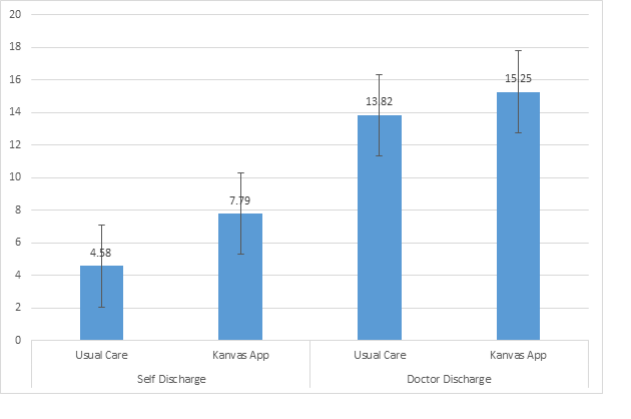
Kept appointments within the usual care and Kanvas app groups by self- versus provider-discharge.

**Figure 5:**
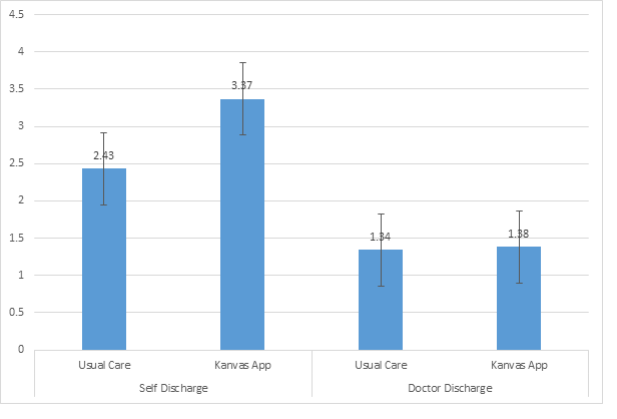
No-show appointments with the usual care and the Kanvas app groups by self- versus provider-discharge.

Figures 4 and 5 present the kept appointments and no-show appointments within the usual care and the Kanvas app groups by self- versus provider-discharge collapsed across both study years. The 2-way ANOVA used to generate Figure 4 indicated a significant interaction between the study group and the discharge type on kept appointments (*F*_1,4122_=14.46; *P*<.001). Post hoc comparisons indicated that the Kanvas app group had a greater number of kept appointments (mean 7.79, SD 0.25) when compared with the usual care group (mean 4.58, SD 0.18) when both groups were self-discharged. The Kanvas app group had a similar number of kept appointments (mean 15.25, SD 0.28) compared with the usual care group (mean 13.82, SD 0.22) when both groups were provider-discharged. The usual care group had more kept appointments when they were provider-discharged compared with the usual care group who were self-discharged, whereas the Kanvas app group had a similar number of kept appointments when self- or provider-discharged. Figure 5 presents the means of no-show appointments by study group and self- versus provider-discharge. The 2-way ANOVA indicated a significant study group-by-discharge type interaction on no-show appointments (*F*_1,4122_=25.09; *P*<.001). The Kanvas app group (mean 1.38, SD 1.17) and the usual care group (mean 1.34, SD 0.08) were similar in the number of no-show appointments when provider-discharged. The number of no-show appointments when provider-discharged was consistently lower than the number of no-show appointments when these 2 groups were self-discharged. When self-discharged, the Kanvas app group had more no-show appointments (mean 3.37, SD 0.09) compared with the usual care group (mean 2.44, SD 0.07).

## Discussion

### Principal Findings

In general, the results support the study hypothesis that physical health patients who choose to receive the phone-based app compared with physical health patients who choose not to receive the phone app exhibit greater adherence to prescribed physical health treatment. Table 1 clearly indicates that during both 2019 and 2020, a greater proportion of patients who received the Kanvas app completed the prescribed therapy (were provider-discharged) when compared with the usual care group who did not receive this app. An interesting observation in Table 1 is the decline between 2019 and 2020 in patients in both the Kanvas app and the usual care groups who were provider-discharged. This decline in the proportion of patients in both study groups between 2019 and 2020 who adhered to their prescribed therapy by being provider-discharged may be attributed to the COVID-19 pandemic–related social distancing and self-isolation recommendations provided by governmental health agencies during 2020. This effect of the COVID-19 pandemic recommendations may have also accounted for the increase in no-show appointments observed in both study groups between 2019 and 2020 (Table 2). Although this increase in no-show appointments between 2019 and 2020 was only statistically significant among the Kanvas app group (1.89 vs 2.89), the usual care group also exhibited a nonsignificant trend in increased no-show appointments between 2019 and 2020 (1.96 vs 2.14).

A further observation based on this table is that both study groups exhibited a similar number of kept appointments and no-show appointments in 2019. By contrast, during 2020, the Kanvas app group exhibited significantly greater kept appointments and no-show appointments when compared with the usual care group. A potential explanation for these findings may be that during the COVID-19 pandemic restrictions, the Kanvas app may have better engaged the patients in this group to schedule more clinic visits, which resulted in more kept appointments with a greater proportion of them adhering to their prescribed therapy and being provider-discharged. In addition, with additional scheduled appointments comes the potential to increase no-show appointments. In other words, patients in the Kanvas app group appear to have been scheduling more appointments and therefore had a greater potential for both kept and no-show appointments.

Figures 4 and 5 clearly indicate that the number of kept and no-show appointments were similar among the group who received the Kanvas app and the usual care group when they completed their course of care and were provider-discharged. These similarities between the 2 groups are to be expected, as all the patients in these 2 groups completed their prescribed course of care with a similar number of prescribed clinic appointments and a similar potential for no-show appointments. The Kanvas app did not appear to influence the number of kept and no-show appointments among patients who completed their prescribed course of care and were provider-discharged. If the patient prematurely terminated their care or was self-discharged, then the patients in this group who received the Kanvas app had significantly more kept appointments than the usual care group (7.79 vs 4.58). An explanation for this is that the Kanvas app group had more scheduled appointments, which may have contributed to the Kanvas app group exhibiting more no-show appointments than the usual care group when both groups were self-discharged (3.37 vs 2.43). The Kanvas app did not appear to affect adherence among patients who completed their prescribed therapy and were provider-discharged. Patients who were self-discharged and received the Kanvas app experienced on average 3.2 more kept appointments, a 70% increase, and 0.94 more no-show appointments than the self-discharged usual care group.

### Comparison With Prior Work

The findings of this study are consistent with those of previous studies and address a number of gaps in the literature. The key finding of this study was that patients who self-discharged and accessed the Kanvas app exhibited greater adherence to their prescribed therapy in the form of keeping scheduled appointments when compared with patients who self-discharged and did not access the Kanvas app. This finding that technology-based health interventions, including phone apps, can increase adherence to prescribed therapies has been reported by previous authors [24,25,27-29]. This study is one of the first to demonstrate the efficacy of a phone app to increase adherence among patients prescribed physical health treatment by attending a chiropractic and rehabilitation clinic. This finding is particularly significant, as the literature indicates that physiotherapy patients are frequently not adherent and do not complete their prescribed therapy [3,4,12].

### Strengths, Limitations, and Future Studies

This study has a number of strengths and limitations that may direct future inquiry in this area. The validity of this study is strengthened by the large sample size collected over multiple clinical sites using EMR as the source of the outcome variables, including kept and no-show appointments and physician versus self-discharge. These data are clinically valid, as billing and reimbursement are based on information stored in EMRs. Although encouraging, these findings must be interpreted cautiously because of a number of methodological limitations. First, participants in the study groups self-selected to download the Kanvas app; thus, patients who were more likely to adhere to their prescribed therapy may also have self-selected to download the Kanvas app. From the findings, it is unclear whether a patient characteristic that predisposed them to adhere to prescribed treatment may have also increased their likelihood of self-selecting to receive the Kanvas app. Data on demographic characteristics of the participants were not collected in this study and may have influenced the decision to self-select one of the study groups. Although generally desirable in clinical studies, the large sample size cultivated in this study increased the likelihood of detecting the statistical significance of small effect sizes. This limitation is tempered by the clinical significance of the Kanvas app group, exhibiting 3.2 more kept appointments and 0.94 more no-show appointments than the usual care group when both groups were self-discharged. Another limitation of this study was that the cost benefit of implementing the Kanvas app was not examined. Although numerous studies have reported the clinical efficacy of technology-based health interventions, including phone apps, few studies have consistently found that these interventions generate revenue or are at least cost neutral while benefiting patients [34,35]. Finally, the findings may have been influenced by governmental recommendations for social distancing and self-isolation in 2020. The influence of these recommendations is evident in the decline in provider-discharge and the increase in no-show appointments observed in both the Kanvas app and usual care groups in 2020 compared with 2019. However, these declines in adherence metrics between 2019 and 2020 were less evident in the Kanvas app group compared with the usual care group. A final limitation to the validity of the findings is that individual patient use of the Kanvas app was not monitored, and if the patient self-selected to download the app, there was no way to monitor the type or duration of interaction the individual had with the app. Similarly, the development of the Kanvas app may have benefited from input from the end users or a feedback loop allowing the user to suggest improvements in the app that may foster long- and short-term engagement [34]. Involving end users in the refinement of health-promoting phone apps may foster engagement, motivation, and autonomy with the app [35,36]. Future studies may wish to address these limitations by randomly assigning patients willing to download the app to study groups who do and do not receive the app. Future refinement of the Kanvas app may consider involving end users in changes to the app. In addition, qualitative methods may be used to determine why patients decided to decline downloading the app and what features of a future app may be appealing to them to increase their adherence to prescribed treatments.

### Conclusions

The findings of this study support the efficacy of the Kanvas app in increasing adherence to prescribed physical health treatment among patients attending a chiropractic and rehabilitation clinic. These benefits of the Kanvas app appear to differentially affect patients who self-discharge, although not measurably affecting provider-discharged patients. Patients who self-discharged and received the Kanvas app exhibited significantly more kept appointments and more no-show appointments than a usual care group that did not receive the Kanvas app.

## Abbreviations

ANOVA: analysis of variance
EMR: electronic medical record
mHealth: mobile health
RCT: randomized controlled trial
